# Efficacy and factors associated with reactivation following intravitreal ranibizumab or conbercept for retinopathy of prematurity

**DOI:** 10.3389/fmed.2026.1754490

**Published:** 2026-02-19

**Authors:** Qiuhui Liu, Jing Li, Jiafen Liu, Suzhen Xie, Ge Mu, Huanhuan Zhao, Jiao Zheng, Xuelin Huang, Jianxun Wang

**Affiliations:** Guangdong Women and Children Hospital, Guangzhou, China

**Keywords:** anti-VEGF (vascular endothelial growth factor), conbercept, ranibizumab, reactivation, ROP (retinopathy of preterm)

## Abstract

**Purpose:**

To compare the efficacy of intravitreal ranibizumab (IVR) and conbercept (IVC) treatment for retinopathy of prematurity (ROP) and to evaluate the risk factors associated with disease reactivation.

**Methods:**

In this retrospective study, the medical records of infants with ROP treated with IVR or IVC from April 2017 to June 2024 at Guangdong Women and Children Hospital were reviewed. The primary outcome measures were reactivation rate, time to reactivation, and factors associated with reactivation.

**Results:**

A total of 294 infants (565 eyes) were included. The reactivation rate was 10.39% (43 of 414 eyes) in the IVR group and 12.58% (19 of 151 eyes) in the IVC group, with no significant difference between the two groups (*p* = 0.46). The mean time to reactivation was similar between the IVR group (8.92 ± 2.01 weeks) and the IVC group (8.78 ± 1.68 weeks) (*p* = 0.84). Multivariate logistic regression analysis identified that a lower postmenstrual age (PMA) at initial treatment (*p* = 0.001) and Zone I ROP (*p* = 0.008) were significant independent risk factor of reactivation. Gestational age, birth weight, sex, and other systemic comorbidities were not significantly associated with reactivation.

**Conclusion:**

Ranibizumab and conbercept are effective for treating ROP. A lower PMA at initial treatment and Zone I ROP were identified as significant risk factors for reactivation. Infants with these characteristics require closer and more prolonged follow-up after anti-VEGF therapy.

## Introduction

Retinopathy of prematurity (ROP) is a multifactorial disease affecting the retinal vasculature of premature infants (1). Despite continuous advances in neonatal and ophthalmic care, ROP remains a leading cause of childhood blindness worldwide (2). The pathogenesis of ROP is critically driven by hypoxia-associated dysregulation of vascular endothelial growth factor (VEGF) (3).

Anti-VEGF agents, including ranibizumab and conbercept, have recently been demonstrated to be effective in treating ROP (4). Compared with retinal laser photocoagulation, intravitreal anti-VEGF injections offer several advantages, including more rapid regression of tunica vasculosa lentis and plus disease, a reduced risk of future myopia, and preservation of peripheral visual field (5). Despite the efficacy of intravitreal anti-VEGF therapy for ROP, reactivation remains a clinical concern. Reported reactivation rates vary considerably across studies, which may be attributable to differences in the type and dose of anti-VEGF agents administered (4). Previous studies have identified both prenatal and postnatal factors as being associated with ROP reactivation (6).

However, direct comparisons of reactivation rates and risk factors between intravitreal ranibizumab (IVR) and intravitreal conbercept (IVC) therapies remain limited. This study aimed to determine and compare the reactivation rates, timing of reactivation, and risk factors associated with disease reactivation following IVR and IVC treatment.

## Methods

The study protocol was approved by the Institutional Review Board and Ethical Committee of Guangdong Women and Children Hospital (Guangzhou, China) and was conducted in accordance with the tenets of the Declaration of Helsinki. Written informed consent was obtained from the parents or guardians of each infant prior to the intravitreal injection.

A retrospective chart review of infants who underwent IVR and IVC treatment for Zone I/II Stage 2/3 + ROP and A-ROP was conducted. The study included infants who presented at the Eye Center of Guangdong Women and Children Hospital from April 2017 to June 2024, with a minimum follow-up to 75 weeks of PMA. All patients were treated with either IVR (0.25 mg/0.025 mL; Lucentis, Genentech Inc.) or IVC (0.25 mg/0.025 mL; Conbercept, Chengdu Kanghong Biotech Co., Ltd.) in this study. The ranibizumab dose of 0.25 mg used in this study is consistent with our institutional protocol. It is important to note that this dose is higher than those investigated in the RAINBOW trial (7). Exclusion criteria comprised: a history of any prior intravitreal anti-VEGF injection, laser photocoagulation, cryotherapy, or other ocular surgeries; and the presence of retinal detachment at presentation. The stage and zone of ROP were classified according to the International Classification of Retinopathy of Prematurity (ICROP) guidelines. Fundus examinations were performed using the RetCam III (Clarity Medical Systems, Pleasanton, CA, United States), and treatment decisions were made by two senior ophthalmologists (S.X. and X.H.). All treated eyes met the treatment criteria outlined by the Early Treatment for Retinopathy of Prematurity (ETROP) Cooperative Group.

Intravitreal injections were administered following established expert panel guidelines. Post-treatment fundus examinations were conducted on day 3, then at 1 week, 2 weeks, and bi-weekly thereafter until complete ROP regression was observed. Subsequently, the examination interval was extended to every 4–8 weeks until 75 weeks of PMA.

A positive anatomic outcome was defined as the regression of plus disease, the disappearance of the ridge and shunt vessels, and the restoration of normal retinal vascular architecture. Reactivation of ROP was defined as the recurrence of acute-phase features, which could range from a new demarcation line to Stage 3 with plus disease, accompanied by renewed vascular dilation and tortuosity. Cases of reactivation were managed with a second anti-VEGF injection, laser photocoagulation, or a combination therapy.

Medical records of the infants were reviewed, including: primary and final outcomes, birth weight (BW), gestational age (GA), postmenstrual age (PMA) at initial treatment, sex ratio, ROP zone and stage, time to reactivation, and systemic comorbidities such as history of patent foramen ovale (PFO), patent ductus arteriosus (PDA), neonatal sepsis, necrotizing enterocolitis (NEC), pneumonia, intracranial hemorrhage (ICH), history of systemic surgery, and multiple birth.

Statistical analyses were performed using SPSS software (version 27.0 for Windows; SPSS Inc., Chicago, IL, United States). Continuous variables are expressed as mean ± standard deviation and were compared using the Student’s *t*-test. Categorical variables are presented as frequencies and percentages, and group differences were assessed using the Chi-square test. To identify factors associated with ROP reactivation, candidate variables were first prescreened using univariate logistic regression (*p* < 0.1 for inclusion). Subsequently, a multivariable logistic regression model was constructed using backward selection to determine independent associations. The results are reported as odds ratios (OR) with corresponding 95% confidence intervals (CI). And Kaplan–Meier survival curves were constructed for the two treatment groups, with non-reactivation as the endpoint. Differences in survival distributions were compared using the Log Rank test. The level of statistical significance was set at *p* < 0.05.

## Results

### Demographic characteristics

We reviewed the medical records of infants with ROP over a 6-year period, and 565 eyes from 294 infants met the inclusion criteria. The demographic characteristics of the infants are summarized in Table 1.

**Table 1:** Demographics and clinical characteristics of infants with ROP.

Characteristics	IVR	IVC	P*
Sum of infants/eyes	217/414	77/151	N/A
Male gender, no (%)	134 (61.75%)	41 (53.25%)	0.191
Birth weight (g)	1064.22 ± 328.02	1134.29 ± 289.71	0.098
Gestational age (weeks)	28.04 ± 2.27	28.60 ± 2.12	0.058
PMA of initial treatment (weeks)	37.88 ± 3.91	37.35 ± 2.99	0.278
ROP characteristics			
Stage 2, no (%)	125 (32.22%)	33 (24.44%)	0.09
Stage 3, no (%)	263 (67.78%)	102 (75.56%)	0.09
Zone 1, no (%)	30 (7.73%)	14 (10.37%)	0.34
Zone 2, no (%)	358 (92.27%)	121 (89.63%)	0.34
A-ROP, no (%)	26 (6.28%)	16 (10.60%)	0.08

* Continuous variables were compared using Student’s *t*-test and categorical variables were compared using the Chi-square test. PMA, postmenstrual age; ROP, retinopathy of prematurity; A-ROP, aggressive ROP; N/A, not applicable.

**Figure 1:**
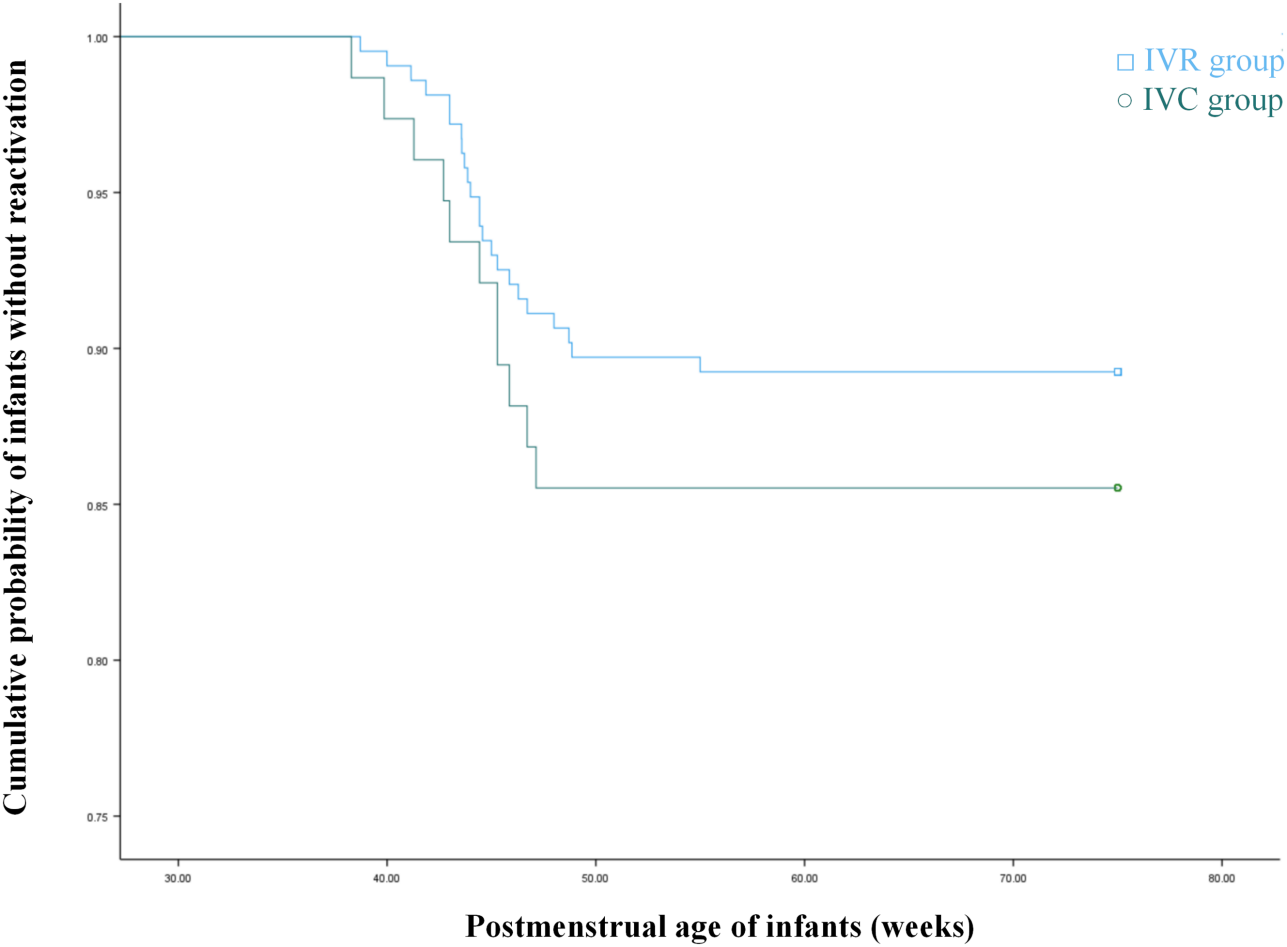
Kaplan–Meier analysis of non-reactivation ROP incidence showed no statistically significant difference between the IVR and IVC groups.

No statistically significant differences were observed between the different anti-VEGF agent groups regarding sex ratio, BW, GA, PMA at initial treatment, or ROP characteristics (Table 1). Salvage laser therapy was administered within 1 week after injection due to aggravated fibrous proliferation in 5 eyes (1.21%) in the ranibizumab group and 3 eyes (1.99%) in the conbercept group (*p* = 0.49). Reactivation of ROP occurred in 43 eyes (10.39%) following intravitreal ranibizumab (IVR) and 19 eyes (12.58%) following intravitreal conbercept (IVC) (*p* = 0.46). The mean interval between injection and reactivation was 8.92 ± 2.01 weeks (range: 5.71 to 13.71 weeks) in the ranibizumab group and 8.78 ± 1.68 weeks (range: 6.43 to 12.14 weeks) in the conbercept group. This injection interval did not differ significantly between the ranibizumab and conbercept groups (*p* = 0.84) (Table 2). In this study, ROP reactivation occurred between 38.29 and 55 weeks of PMA (38.72–55 weeks in the IVR group and 38.29–47.14 weeks in the IVC group). The recurrence-free survival of the two groups, as assessed by the Kaplan–Meier curve (Figure 1), did not show a statistically significant difference (*p* = 0.372).

**Table 2:** Outcome after initial treatment.

Outcome	IVR	IVC	P*
No. of eyes	414	151	N/A
Rate of salvage lase therapy within 1 week, no (%)	5 (1.21%)	3 (1.99%)	0.49
Rate of reactivation, no (%)	43 (10.39%)	19 (12.58%)	0.46
The interval between injection and reactivation, weeks	8.92 ± 2.01	8.78 ± 1.68	0.84

* Continuous variables were compared using Student’s *t*-test and categorical variables were compared using the Chi-square test. N/A, not applicable.

### Factors associated with reactivation

To identify factors associated with ROP reactivation, potential risk factors were analyzed using binary logistic regression. The variables considered included GA, PMA at initial treatment, BW, gender, ROP zone, history of PFO, PDA, neonatal sepsis, NEC, pneumonia, ICH, history of systemic surgery, and multiple birth.

Multivariate regression analysis identified only two significant independent risk factors for ROP reactivation: PMA at initial treatment (*p* = 0.001) and Zone I ROP (*p* = 0.008). Specifically, each additional week in PMA at initial treatment was associated with a 0.6-fold reduction in the odds of reactivation (Odds Ratio [OR] = 0.631; *p* = 0.001). Furthermore, Zone I ROP was associated with a 4.5-fold increase in the odds of reactivation compared to Zone II ROP (OR = 4.546; *p* = 0.008). Aggressive ROP (A-ROP) (OR = 3.009, *p* = 0.106) and a history of systemic surgery (OR = 2.293, *p* = 0.095) showed a trend being associated with increased risk but did not reach statistical significance in this study. GA, BW, gender, history of PFO, PDA, neonatal sepsis, NEC, pneumonia, ICH, and multiple birth were not significantly associated with ROP reactivation (*p* > 0.05 for all) (Table 3).

**Table 3:** Univariate and multivariate logistic regression analysis of risk factors for ROP reactivation.

Risk factor	Univariate			Multivariate		
	OR	95%CI	*P*	OR	95%CI	*P*
Gestational age (weeks)	0.685	0.559, 0.84	0.001	0.878	0.592, 1.302	0.519
PMA of initial treatment (weeks)	0.605	0.492, 0.746	0.001	0.631	0.499, 0.798	0.001
Birth weight (g)	0.998	0.996, 0.999	0.002	0.999	0.996, 1.001	0.315
Female gender	0.463	0.208, 1.031	0.059	0.532	0.213, 1.332	0.178
Zone I ROP	6.729	2.617, 17.302	0.001	4.546	1.475, 14.008	0.008
A-ROP	2.424	0.832, 7.060	0.105	3.009	0.79, 11.467	0.106
Patent foramen ovale	1.587	0.587, 4.292	0.363			
Patent ductus arteriosus	1.505	0.732, 3.092	0.266			
History of neonatal sepsis	1.565	0.718, 3.407	0.260			
History of necrotizing enterocolitis	2.779	1.277, 6.046	0.01	1.11	0.421, 2.926	0.833
History of pneumonia	1.723	0.772, 3.844	0.184			
History of intracranial hemorrhage	1.668	0.811, 3.430	0.164			
History of systemic surgery	2.095	0.932, 4.710	0.074	2.293	0.865, 6.077	0.095
Multiple Birth	1.097	0.486, 2.474	0.823			

OR, odds ratio; CI, confidence interval; PMA, postmenstrual age; ROP, retinopathy of prematurity; A-ROP, aggressive ROP.

## Discussion

The main findings of this study were as follows. First, both IVR and IVC are effective treatments for Type 1 ROP. The reactivation rate after IVR treatment was 10.39% (43 of 414 eyes); and the IVC group had a reactivation rate of 12.58% (19 of 151 eyes). There is no significant difference between the two groups (*p* = 0.46). Second, the mean interval to reactivation was 8.92 ± 2.01 weeks in the IVR group, and 8.78 ± 1.68 weeks in the IVC group. The difference between groups did not reach statistical significance. Finally, a lower PMA at initial treatment and Zone I ROP were identified as significant risk factors for recurrent ROP.

Recently, anti-VEGF agents have emerged as an effective treatment for ROP. Compared with laser photocoagulation, anti-VEGF treatment can preserve the peripheral retina, avoid postoperative visual field defects and reduce the incidence of high myopia (8). In addition, anti-VEGF treatment has obvious advantages for children with A-ROP, zone I disease, vitreous opacity, retinal hemorrhage, iris neovascularization, or poor general condition that cannot tolerate general anesthesia (9).

Despite these benefits, reactivation following anti-VEGF monotherapy has been increasingly reported. The reported reactivation rate after ranibizumab treatment ranges from 2.3 to 83% across studies (10–13). In the RAINBOW study, 22 eyes (15%) in the ranibizumab 0.2 mg group and 26 eyes (17%) in the ranibizumab 0.1 mg group received additional treatment due to ROP reactivation (7). In our study, the reactivation rate in the ranibizumab group was lower than that in the ranibizumab 0.2 mg group in RAINBOW study. Reports on the reactivation rate following conbercpet treatment are relatively limited. Previous studies indicate reactivation rates following conbercept injection ranging from 15 to 16.7%. Jin et al. reported that 15% (3/20) of eyes in the conbercept 0.25 mg group required reinjection (14). Similarly, in the investigation conducted by Wu et al., reactivation was observed in 16.7% (10/60) of eyes among pediatric patients receiving the same dose (15). In our study, the reactivation rate in the conbercept group was slightly lower than that reported in the aforementioned studies. This discrepancy may be attributed to heterogeneity in the baseline characteristics and retinopathy of prematurity (ROP) features among the enrolled subjects across these studies. The RAINBOW study enrolled a higher proportion of Zone I ROP cases (28 out of 70) in the ranibizumab 0.2 mg group (5). In contrast, the proportion of Zone I ROP in the ranibizumab group of our study was 7.73%, which is considerably lower than that reported in the RAINBOW study. Additionally, in our study, the PMA at initial treatment was greater than that in both Wu’s study and the RAINBOW study. Furthermore, unlike the dosage used in the RAINBOW study, patients in the ranibizumab group of our study received intravitreal injections of 0.25 mg ranibizumab.

The reactivation interval of ROP varies greatly based on the type and dose of anti-VEGF injection. The literature reports varying average reactivation intervals: 6–13 weeks for ranibizumab (16–18), 6–16 weeks for aflibercept (19–23), and 4–10 weeks for conbercept (20, 24). In our study, the mean injection interval was 8.92 + 2.01 weeks in the ranibizumab group and 8.78 ± 1.68 weeks in the conbercept group. Previous investigations have indicated that reactivation appear earlier with ranibizumab than with bevacizumab or aflibercept (25). Researchers have postulated that this may be attributable to the shorter intraocular half-life of ranibizumab compared to aflibercept and bevacizumab, owing to its smaller molecular size and differential binding affinity. Although conbercept has a larger molecular weight and higher VEGF-binding activity than ranibizumab (26), the injection intervals between the two groups in our study were nearly identical. This may be explained by inter-individual variations among the enrolled patients and the relatively limited sample size in the conbercept group. In our study, ROP reactivation might occur until 55 weeks of PMA. Notably, the BEAT-ROP trial has also demonstrated that ROP reactivation can occur as late as 65 weeks PMA (27), underscoring the critical importance of close and prolonged follow-up after anti-VEGF injections.

Multiple risk factors have been identified for reactivation of ROP after anti-VEGF treatment, including smaller GA (28), lower BW (29), early PMA at treatment (30), history of NEC, history of intubation (31), anemia (31), sepsis (31), periventricular leukomalacia (32), PDA (32), low Apgar scores (33). Ling et al. found that independent risk factors of reactivation included an early PMA at initial treatment, Zone I disease, low Apgar score, and multiple births (33). While Huang et al. indicated that GA, Zone I ROP and A-ROP were independent factors for reactivation (28).

In our study, the confirmed independent risk factors for ROP reactivation included early PMA at initial treatment and Zone I ROP. A lower PMA at initial treatment suggests greater disease severity and more serious retinopathy, requiring earlier therapy. Moreover, anti-VEGF treatment administered before 35 weeks PMA may be inadequate to block VEGF production during Phase 2 ROP (approximately 31–44 weeks PMA), as evidenced by the BEAT-ROP study (27). Further study is needed to determine the required dose and the type of anti-VEGF agents in infants with lower PMA who require treatment. In contrast, we found that GA and BW had little correlation with reactivation of ROP, which is consistent with the findings of some studies.

The present study has several limitations that must be considered. First, it is a single-center, retrospective study. Consequently, detailed data on systemic outcomes (e.g., duration of respiratory support, survival post-discharge, evidence of systemic anti-VEGF exposure) and long-term post-discharge visual and neurodevelopmental outcomes were not systematically collected. Future prospective research with larger cohorts, extended follow-up, and comprehensive systemic and developmental assessments is needed for a more holistic evaluation of anti-VEGF therapy in ROP. Second, fundus photographs were not quantitatively analyzed in this study. Future research should incorporate detailed analysis of retinal vascular features to better understand their association with ROP reactivation.

Nonetheless, our findings support the efficacy of IVR and IVC for ROP in infants. Infants with a lower PMA at initial treatment and Zone I ROP need closer follow-up after anti-VEGF injection due to the high risk of reactivation.

## Data Availability

The original contributions presented in the study are included in the article/supplementary material, further inquiries can be directed to the corresponding author.
